# Collaboration, supervision and patient safety in the era of COVID-19: an analysis of medical wards and ICU

**DOI:** 10.1007/s11845-021-02693-1

**Published:** 2021-07-04

**Authors:** Marc Lincoln, Ahmed Gabr, Cormac Kennedy, Catherine Murphy, Aileen Patterson, Enda O’Connor, Martina Hennessy

**Affiliations:** 1 Department of Therapeutics and Clinical Pharmacology, St James’s Hospital, Dublin, Ireland; 2 Central Research Facility, St James’s Hospital, Dublin, Ireland; 3 School of Medicine, Trinity College Dublin, Dublin, Ireland; 4 Intensive Care Unit, St James’s Hospital, Dublin, Ireland

**Keywords:** Collaboration, COVID-19, Patient safety, Supervision, Ward-based medical care

## Abstract

**Aims:**

COVID-19 resulted in significant changes across medical wards and ICU in St James’s Hospital Dublin. This included the implementation of ward-based medical teams (WBMT). The purpose of this study was to identify how these structural changes affected inter-professional collaboration, supervision and patient safety.

**Methods:**

Questionnaires were distributed to doctors working on medical wards and ICU at the height of the first wave of COVID-19. The sense of collaboration, patient safety and supervision were assessed.

**Results:**

Fifty-three doctors took part in the study. Thirty-three (62%) felt that collaboration was better than normal. Forty-six (87%) of participants described supervision as “good” or “excellent”. Thirty-one out of 40 participants (77%) felt that patient safety was better than normal.

**Discussion:**

Implementation of WBMT may result in improved sense of collaboration, supervision and patient safety during COVID-19; however, the increased sense of solidarity and comradery felt during the initial surge make drawing these conclusions challenging.

## Introduction

The first COVID-19 surge resulted in significant changes across the medical wards and the intensive care unit (ICU) in St James’s hospital, Dublin.

On medical wards prior to the crisis, newly admitted patients were spread across various wards. During COVID-19, although the make-up of medical teams remained the same, they were distributed using a Ward Based Medical Teams system (WBMT) with each having a “home” ward in order to reduce virus transmission. In the ICU, the department was split into a COVID and a non-COVID side and intensivists were assigned to each.

As a result of these structural alterations, inter-professional collaboration, senior supervision and patient safety may thus be impacted. The aim of this study was to identify how these structural changes on medical wards and ICU affected the above parameters during the first surge of COVID-19.

## Methods

This study was a single-centre survey conducted between April 16th and May 15th, 2020, among doctors on both ICU and medical wards at St James’s Hospital (SJH), Dublin. Questionnaires were distributed to doctors participating at these locations during the height of COVID-19 to assess the levels of collaboration, supervision and sense of patient safety.

Participants were asked to compare their sense of collaboration prior to and during and the pandemic. They were assessed using a 5-point Likert scale where a rank of “1” = much worse, “3” = similar and “5” = much better. Staff were also given the option of adding general comments.

Similarly, supervision was assessed using a 5-point non-numerical ordinal scale from “insufficient” to “same” to “excellent”. This part of the study did not directly compare experiences prior to and during COVID.

Lastly, participants were asked to compare their sense of patient safety during and prior to the pandemic using a 10-point Likert scale where “1” indicated “much less safe”, “5” indicated “as safe” and “10” indicated “better”.

## Results

### Collaboration

**Fig. 1:**
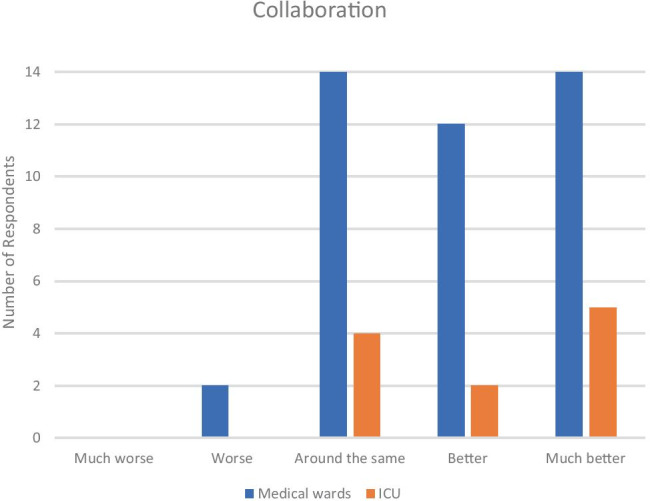
Sense of collaboration on medical wards and ICU

Thirty-three out of 53 doctors on both medical wards and ICU had found that collaboration during the pandemic was “better” or “much better” (Fig. 1). These responses were evenly spread across all hospital grades. In the comments section, the general theme was that collaboration was improved through “improved work patterns” and “increased efficiency”.

### Supervision

Forty-six out of 53 respondents felt that supervision was “good” or “excellent”. Eight respondents reported supervision to be “at times sufficient” or “just sufficient”. These sentiments were shared among all hospital grades.

### Patient safety

Thirty-one out of 40 respondents reported a score of “6” or better with a median score of 8. This was shared among all hospital grades.

## Discussion

Our study demonstrated that, by implementing a WBMT at the height of the first wave of COVID-19, the senses of collaboration and patient safety were felt to be overall better in comparison to prepandemic where doctors had to migrate to multiple wards. The level of supervision was found to be overall good or excellent.

The improved sense of collaboration may be attributed to better communication between healthcare professionals. This was consistent with previous studies which showed that transformation to WBMT has resulted in improved rapport, overall less number of bleeps and interruptions, teamwork between healthcare professional and doctors’ accessibility [1–3]. In contrast to a previous study where less sense of supervision was highlighted as a potential drawback of the WBMT system, our findings showed increased sense of supervision [4]. We speculate that cancellations of elective surgeries, outpatient clinics, and restrictions of annual leaves may have contributed to the availability of more senior staff to allocate more time to ward care. In addition, the increased sense of patient safety may simply be attributed to the increased supervision.

The main strength of our study is that it was performed during the height of the first wave of the pandemic and thus is a contemporary reflection of doctors’ experiences. However, the unique sense of comradery and solidarity felt during this initial surge makes drawing solid conclusions and attributing our positive findings to the development of WBMT challenging. In addition, we did not directly compare supervision before and during the switch to WBMT and therefore cannot state that the sense of supervision as actually better than prior to WBMT.
